# Targeting Hormone-Related Pathways to Improve Grain Yield in Rice: A Chemical Approach

**DOI:** 10.1371/journal.pone.0131213

**Published:** 2015-06-22

**Authors:** Hiroaki Tamaki, Maria Reguera, Yasser M. Abdel-Tawab, Yumiko Takebayashi, Hiroyuki Kasahara, Eduardo Blumwald

**Affiliations:** 1 Department of Plant Sciences, University of California Davis, Davis, California 95616, United States of America; 2 Health and Crop Sciences Research Laboratory, Sumitomo Chemical Co. Ltd., Hyogo 665–8555, Japan; 3 RIKEN Center for Sustainable Resource Science, Yokohama, Kanagawa 230–0045, Japan; Texas Tech University, UNITED STATES

## Abstract

Sink/source relationships, regulating the mobilization of stored carbohydrates from the vegetative tissues to the grains, are of key importance for grain filling and grain yield. We used different inhibitors of plant hormone action to assess their effects on grain yield and on the expression of hormone-associated genes. Among the tested chemicals, 2-indol-3-yl-4-oxo-4-phenylbutanoic acid (PEO-IAA; antagonist of auxin receptor), nordihydroguaiaretic acid (NDGA; abscisic acid (ABA) biosynthesis inhibitor), and 2-aminoisobutyric acid (AIB; ethylene biosynthesis inhibitor) improved grain yield in a concentration dependent manner. These effects were also dependent on the plant developmental stage. NDGA and AIB treatments induced an increase in photosynthesis in flag leaves concomitant to the increments of starch content in flag leaves and grains. NDGA inhibited the expression of ABA-responsive gene, but did not significantly decrease ABA content. Instead, NDGA significantly decreased jasmonic acid and jasmonic acid-isoleucine. Our results support the notion that the specific inhibition of jasmonic acid and ethylene biosynthesis resulted in grain yield increase in rice.

## Introduction

Rice is one of the most important food crops worldwide, and an increased interest in developing high grain-yielding cultivars have led to the development of new varieties using conventional breeding programs [1]. Grain filling and consequently Grain Yield (GY) are dependent on plant source/sink relationships, where the carbohydrates stored during pre-anthesis are mobilized from the vegetative tissues to the grains. Several genes associated with GY improvement have been identified by QTL analysis [2–5]. Most of the genes identified have been functionally associated with sink strengthening and only in the case of the gene *THOUSAND-GRAIN WEIGHT 6* (*TGW6*), a source reinforcement effect has been described [4]. The mechanisms mediating source strengthening that result in sink improvement are still largely unknown.

Plant hormones and their interactions have been shown to play pivotal roles on plant growth and development [6–8]. For instance, ABA plays important roles regulating seed maturation and seed dormancy counteracting with gibberellic acid (GA), brassinosteroids (BRs) and ethylene [9]. Also, ABA stimulates Ethylene production to let leaves abscise [10]. Cytokinins (CK) are well known key regulators of plant growth controlling shoot meristematic activity and cell differentiation in the root meristem [11, 12]. On the other hand, auxins are regulated temporally and spatially to control developmental processes such as organogenesis, embryogenesis and tropism [13]. The antagonistic interaction between auxins and CK is determinant of root and shoot cell differentiation and division, controlling growth and morphogenesis [14]. Interestingly, an induction of ABA synthesis driven by jasmonic acid (JA) has also been proposed [15]. Jasmonic acid (JA) has been mainly related to plant defense responses although a role in development independent of the pathogenesis-related response has also been described [16]. The constitutive synthesis of methyl jasmonate (MeJA) resulted in increased contents of ABA leading to GY penalties in rice. Thus, plant hormones act through a complex network that involves the interaction with other hormones under the fine tune control of master regulators (such as transcription factors) to regulate plant growth, development and more specifically, sink and source relationships [7, 8]. To differentiate specific plant hormone mechanisms, the use of reporters of hormone-specific responsive genes was proposed [17]. Also, the identification of plant hormone-responsive rice genes will be useful to detect and analyze the specific signaling pathways related to each plant hormone [18].

Several hormone inhibitors can improve plant growth only when applied during the late reproductive phase (post-anthesis stage). For instance, paclobutrazol (PBZ), a gibberellic acid (GA) biosynthesis inhibitor that primarily targets the *ent*-kaurene oxidase [19, 20], was applied two weeks after rice panicle initiation, an improvement in GY was achieved [21]. The application of the antagonists of ethylene receptors, such as 1-methylcyclopropane (1-MCP) or 3-cyclopropyr-1-enyl-propanoic acid sodium salt (CPAS), resulted in increased GY in wheat and soybean [22, 23]. NDGA has been used as ABA biosynthesis inhibitor to prevent fruit maturation in tomato [24]. Also, the application of aminoethoxyvinylglycine (AVG) decreases ethylene production to reduce pre-harvest fruit drop in apple ([25] and references therein).

Here, we tested the effects of chemicals that inhibit the biosynthesis, perception, or signaling of plant hormones, and characterize their effects on GY in rice. Among the tested chemicals, we observed that those that inhibit the biosynthesis of GA, BR or auxin negatively affected GY, while the use of inhibitors affecting auxin perception, JA and ethylene biosynthesis improved GY.

## Materials and Methods

### Plant material and growth conditions

Rice seeds (*Oryza sativa* L. ssp. *japonica* cv. Kitaake) were germinated on moist paper for 1 week (28°C in the dark). Seedlings were transplanted into 8 L pots (2 plants per pot), using soil harvested in California rice field (capay series, 38°32′23.93′′N,121°48′30.81′′W, shredded and steamed for 1.5 h to eradicate soil pathogens). Greenhouse conditions were 12 h, 30°C (day) / 12 h, 20°C (night). Plants were fertilized using 50% N:P:K (20:10:20) (Peters professional) and 50% ammonium sulphate (VIKING SHIP). Total nitrogen added was 0.8 g/pot, every 2 weeks until panicle initiation.

### Chemical treatment

The application of plant hormone inhibitors was done by spraying the aerial part of the rice plants using different concentrations of the chemicals (S1 Table) at two developmental stages: pre-anthesis (just before heading stage) and/or post-anthesis (2 weeks after flowering, during grain filling stage). All spraying solutions contained 0.1% dimethyl sulfoxide (DMSO; Sigma-Aldrich, St. Louis, MO) and 0.05% Tween20 (Sigma-Aldrich, St. Louis, MO) to allow chemical penetration into the plant tissue. Untreated control (UTC) plants were sprayed using the same solution without plant hormone inhibitors.

### Gas exchange measurements

Rates of CO2 assimilation were determined in flag leaves of rice plants under same developmental stage using the portable gas exchange system LI-COR 6400–40 (Li-COR Inc. Lincoln, NE, USA). The leaf cuvette was set at photosynthetic photon flux density (PPFD) of 1,500 μmol.m^-2^.s^-1^, 50–60% relative humidity and 29°C of block temperature. Photosynthesis activity and stomata conductance were determined after 2, 9 and 16 days after spray and respiration was estimated by using the equation previously described [26].

### Quantitative PCR analysis (qPCR)

For gene expression analysis, total RNA was extracted from the flag leaves using RNeasyMini kit (Qiagen, Valencia, CA). The quality of RNA was determined using Nanodrop ND-1000. First strand cDNA was synthesized from 1 μg of total RNA using QuantiTect Reverse Transcription kit (Qiagen). Quantitative PCR was performed on the StepOnePlus (Applied Biosystems, Foster City, CA), using SYBR GREEN. The 2^−ΔΔCT^ method [27] was used to normalize and calibrate transcript values relative to the endogenous rice transcription elongation factor (TEF) gene. Six biological replicates were used for the expression analysis. The primer sets used for amplifying different target genes are shown in S5 Table.

### Starch and sugar quantification

Flag leaves and immature grains were sampled 2 days after spray at pre-anthesis stage or post-anthesis stage, and immediately frozen in liquid-N. Mature grains were harvested at the end of the experiment. The frozen samples and mature grains were freeze-dried, and 10 mg of tissue powder was used for the soluble sugar extraction as previously described [28]. Separation of sugars was performed with water as a mobile phase flowing at 0.6 ml min-1 using an Aminex HPX-87C column (300 mm × 7.8 mm; Bio Rad Laboratories, Hercules, CA, USA) which was preceded by a micro-guard cartridge (Carbo-C, pH range 5–9, 30 mm × 4.6 mm; Bio Rad Laboratories, Hercules, CA, USA) and maintained at 80°C. 10 μl extract was injected by an auto-sampler and sugars were detected using a refractive index detector (Agilent G1362A) with Agilent HPLC 1100 series. Starch was quantified as previously described [8].

### Quantification of plant hormones

Plant hormones were quantified using an Agilent 1200 series rapid resolution liquid chromatography and 6420 Triple Quadrupole LCMS system (Agilent). Chromatography was performed on a ZORBAX Eclipse XDB-C18 column (1.8 μm, 2.1 × 50 mm). d2-GA1, d2-GA4, d5-tZ, d3-DHZ, d6-iP, d5-tZR, and d6-iPR obtained from OlChemim, d6-ABA obtained from Icon Isotopes, d2-IAA and d6-SA obtained from Sigma-Aldrich, d2-JA obtained from Tokyo Kasei, and synthesized 13C6-JA-Ile [29] were used as internal standards. Two days after spray of NDGA 0.12 mM at pre-anthesis stage, flag leaves were collected in the morning and immediately frozen in liquid N2. The frozen samples (about 200 mg in fresh weight) were freeze-dried, pulverized with 5-mm zirconia beads by tissue lyzer (QIAGEN), and then extracted in 10 volume of extraction solvent (80% acetonitrile with 1% acetic acid) containing internal standards for 1 h. Extracts were centrifuged at 4°C,14,000 x *g*, 10 min, and the supernatant was collected. This procedure was repeated once without internal standards, and acetonitrile was removed in a Speed Vac (Thermo Fisher). Acidic water extracts were loaded onto an Oasis HLB extraction cartridge (30 mg, 1mL; Waters) and washed with 1 mL of water containing 1% acetic acid to segregate high polar impurities. Plant hormones were eluted with 2 mL of 80% acetonitrile containing 1% acetic acid, and the methanol in this eluent was removed in a Speed Vac. Acidic water extracts were loaded onto an Oasis MCX extraction cartridge (30 mg, 1 mL). After washing with 1 mL of water containing 1% acetic acid, acidic and neutral compounds were eluted with 2 mL of acetonitrile (AN fraction). After washing with water containing 5% (v/v) ammonia solution (28% as NH3), basic compounds were eluted with 2 mL of 60% (v/v) acetonitrile containing 5% (v/v) ammonia solution (28% as NH3). After drying these basic fractions, 30 μL of water containing 1% acetic acid was added and the basic hormones (tZ and iP) in these fractions were analyzed by LC-MS/MS. Subfractions (2.5%) of the AN fractions were collected and prepared as basic fractions to analyze for SA. The remaining 97.5% of the AN fractions were concentrated to acidic water in the Speed Vac to remove acetonitrile and loaded onto Oasis WAX extraction cartridges (30 mg, 1 mL). After washing with 1mL of water containing 1% acetic acid, neutral compounds were eluted with 2 mL of acetonitrile, and acidic compounds were eluted with 2 mL of 80% acetonitrile containing 1% acetic acid. After concentrating these acidic fractions to dryness, 30 μL of water containing 1% acetic acid was added and acidic hormones (IAA, ABA, JA, JA-Ile, GA1, and GA4) in these fractions were analyzed. LC conditions and parameters for LC-ESI-MS/MS analysis are described in S2 and S3 Tables.

### Statistical analysis

The JMP (ver.8.0) statistical package (SAS Institute, Cary, NC) was used for statistical analyses. Three-way ANOVA (level of significance P <0.05) was used to test the effect of chemicals on phenotype. LSMeans t-test was used to compare means by treatment at a probability level of 5%. Levels of significance are represented by asterisks as follows: * indicating significance at *P ≤ 0*.*05*. The experiments were based on randomized complete block design, using four–six replicates.

## Results

### Effects of plant hormone inhibitors on plant biomass

Eight different hormone inhibitors (S1 Table) and three different concentrations of each inhibitor were used to study the effects of hormone regulation on GY in rice. The inhibitor concentrations used were based on the effective concentrations previously described [30–37]

Plants treated at pre-anthesis with the highest and intermediate concentrations of all the hormone inhibitors tested, and the lowest concentrations of 2-aminoisobutyric acid (AIB), silver nitrate (AgNO3), and 2-indol-3-yl-4-oxo-4-phenylbutanoic acid (PEO-IAA), induced a reduction in shoot dry weight (SDW) and/or GY (Table 1). Highest concentrations of all tested chemicals decreased plant height and number of tillers (S1 Fig), and increased white aborted seeds (S2 Fig). The lowest concentration of nordihydroguaiaretic acid (NDGA) significantly improved SDW and GY, as compared to the untreated control (UTC) plants (Table 1 and S4 Table).

**Table 1:** Effects of plant hormone inhibitors applied at pre-anthesis on the shoot dry weight and grain yield.

Primary target for inhibition	Chemicals	mM	Shoot dry weight (g plant^-1^)	Grain yield (g plant^-1^)
-	UTC	-	17.0 ± 0.7	28.1±0.5
ABA synthesis	NDGA	0.12	20.8 ± 1.1 *	33.9 ± 2.0 *
		0.6	13.7 ± 0.4 *	23.5 ± 0.7 *
		3	13.4 ± 0.5 *	23.5 ± 0.7 *
GA synthesis	Paclobutrazol	0.016	13.9 ± 0.7 *	25.3 ± 1.8
		0.08	13.2 ± 0.9 *	22.5 ± 0.8 *
		0.4	11.9 ± 0.7 *	21.3 ± 1.5 *
BR synthesis	Imazalil	0.04	14.3 ± 1.3	24.3 ± 2.4
		0.2	13.8 ± 0.9 *	22.8 ± 1.2 *
		1	13.4 ± 0.5 *	19.7 ± 1.2 *
BR synthesis	Propiconazole	0.04	15.1 ± 1.0	26.9 ± 2.1
		0.2	13.5 ± 0.7 *	23.9 ± 1.1 *
		1	13.4 ± 0.7 *	25.5 ± 1.9
Auxin synthesis	L-AOPP	0.04	14.1 ± 1.4	23.8 ± 2.5
		0.2	13.8 ± 0.5 *	22.9 ± 0.7 *
		1	12.5 ± 1.3 *	23.3 ± 1.8 *
Auxin perception	PEO-IAA	0.04	15.2 ± 0.5 *	26.4 ± 1.8
		0.2	13.6 ± 0.7 *	26.1 ± 2.1
		1	13.7 ± 0.8 *	22.6 ± 0.8 *
Ethylene synthesis	AIB	0.2	14.0 ± 0.8 *	24.2 ± 2.0
		1	13.8 ± 0.7 *	23.1 ± 1.7 *
		5	14.7 ± 1.4	22.3 ± 2.2 *
Ethylene perception	AgNO_3_	0.12	14.3 ± 0.8 *	23.8 ± 0.8 *
		0.6	14.2 ± 0.6 *	21.8 ± 1.8 *
		3	12.7 ± 0.7 *	13.6 ± 0.8 *

Values are Mean ± SE (n = 6).

* indicates significance at *P ≤ 0*.*05*.

When the hormone inhibitors were applied at both pre- and post-anthesis stages, they induced a decrease in SDW and/or GY with exception of PEO-IAA, AIB and NDGA (Table 2). Similar to plants treated with chemicals at only pre-anthesis, highest or intermediate concentrations of all tested chemicals increased white aborted seeds (S3 Fig). It has been shown that the loss of indole-3-acetic acid (IAA)-glucose hydrolase function, mediating the formation of active-auxin from conjugated-auxin, improved grain yield [4]. Consistent with this work, when PEO-IAA was applied at pre- and post-anthesis stages, the lowest concentration significantly improved GY (Table 2). When the lowest concentration of AIB was applied at the pre- and post-anthesis stages, no changes in SDW or GY were observed. Nonetheless, significant improvements on SDW and GY were seen when AIB was applied exclusively at the post-anthesis stage (S4 Table).

**Table 2:** Effects of plant hormone inhibitors applied at both pre-anthesis and post-anthesis on the biomass production and grain yield.

Primary target for inhibition	Chemicals	mM	Shoot dry weight (g plant^-1^)	Grain yield (g plant^-1^)
-	UTC		17.8 ± 1.4	25.7 ± 0.3
ABA synthesis	NDGA	0.12	18.1 ± 1.4	30.5 ± 1.8 *
		0.6	13.8 ± 0.9 *	21.9 ± 1.4 *
		3	14.0 ± 0.2 *	23.1 ± 0.9 *
GA synthesis	Paclobutrazol	0.016	14.3 ± 1.2	24.8 ± 1.8
		0.08	14.5 ± 0.4 *	25.2 ± 0.9
		0.4	13.5 ± 0.9 *	22.1 ± 0.9 *
BR synthesis	Imazalil	0.04	14.4 ± 0.8 *	25.3 ± 2
		0.2	14.1 ± 0.9 *	22.3 ± 1.4 *
		1	13.5 z± 0.8 *	22.0 ± 1.9
BR synthesis	Propiconazole	0.04	14.8 ± 0.5 *	25.8 ± 1.4
		0.2	15.2 ± 1.5	26.7 ± 1.3
		1	13.7 ± 0.7 *	22.2 ± 1.9
Auxin synthesis	L-AOPP	0.04	15.3 ± 1.3	21.9 ± 1.4 *
		0.2	14.2 ± 1.0 *	23.1 ± 1.8 *
		1	13.9 ± 1.0 *	23.6 ± 2.1
Auxin perception	PEO-IAA	0.04	17.1 ± 1.4	29.9 ± 1.9 *
		0.2	14.2 ± 1.0	23.1 ± 1.8
		1	14.7 ± 0.6	22.6 ± 1.2 *
Ethylene synthesis	AIB	0.2	15.7 ± 0.4	26.4 ± 1.4
		1	14.8 ± 0.8 *	24.4 ± 0.9
		5	15.1 ± 1.0 *	24.7 ± 1.1
Ethylene perception	AgNO_3_	0.12	13.4 ± 0.5 *	21.4 ±0.7
		0.6	15.5 ± 0.8	21.5 ± 0.9 *
		3	13.7 ± 0.6 *	12.7 ± 0.8 *

Values are Mean ± SE (n = 6).

* indicates significance at *P ≤ 0*.*05*.

### Changes in gene expression associated with alterations in hormone contents and/or signaling

Changes in hormone homeostasis in plants influence many aspects of plant growth and development [7, 38, 39]. To determine the action of the different inhibitors used in this work, we evaluated the changes in the expression of genes associated with the biosynthesis, perception or signaling of each hormone as they were affected by the chemical treatments.

Because of the extensive crosstalk between the hormone-dependent signaling pathways, it is difficult to ascribe specific inhibitory roles to the different compounds used. The expression of hormone-specific marker genes [18] was assessed for each treatment. For each chemical, we selected and evaluated the ABA-specific responsive genes *Rice* *b* *asic-region leucine-* *zip* *per 23* (*OsbZIP23*) and *L* *ate-* *e* *mbryogenesis* *a* *bundant 3* (*LEA3*) [18, 40, 41], the GA responsive gene *Gibberellin 20 oxidase 2* (*GA20ox2*) [2, 8], the BR responsive *Dw* *ar* *f4* (*DWF4*) and the *B* *rassinosteroid* *u* *p-regulated* *1* (*BU1*) [42–44], the auxin-specific responsive genes *Rice* *i* *ndole-3-* *a* *cetic* *a* *cid 4* (*OsIAA4*) and *LOC_Os11g32110* [18, 45], and the ethylene-specific responsive genes *Rice ethylene receptor 2* (*OsETR2*) and *Rice* *e* *thylene* *r* *esponse* *s* *ensor* *1* (*OsERS1*) [18, 46]. The qRT-PCR data showed that the expression of *OsbZIP23* and *LEA3* were down-regulated with increasing NDGA concentrations (Table 3). It has been previously shown that the expression of the GA-associated gene *GA20ox2* was inhibited by exogenous GA application and up-regulated by the GA inhibitors uniconazole or paclobutrazol [2]. Here, the expression of *GA20ox2* was up-regulated with increasing NDGA concentrations, while the expression of *OsIAA4* decreased (Table 3). Taken together, the results suggest that the lowest NDGA concentrations preferentially inhibited ABA signaling-related transcripts.

**Table 3:** Changes in the relative expression of rice hormone-responsive genes after inhibitor application.

		ABA responsive genes		GA responsive gene	BR responsive genes		Auxin responsive genes		Ethylene responsive genes	
Chemicals	mM	*OsbZIP23*	*LEA3*	*GA20ox2*	*DWF4*	*BU1*	*OsIAA4*	*LOC_Os11g32110*	*OsETR2*	*OsERS1*
NDGA	0.12	0.9 ± 0.07	**0.8 ± 0.09** *	1.3 ± 0.08	1.2 ± 0.10	-	0.9 ± 0.20	-	1.0 ± 0.10	-
	0.6	**0.7 ± 0.05** *	**0.8 ± 0.07** *	**1.7 ± 0.25** *	1.2 ± 0.13	-	0.9 ± 0.12	-	0.9 ± 0.14	-
	3	**0.4 ± 0.04** *	**0.6 ± 0.09** *	**1.8 ± 0.35** *	1.0 ± 0.25	-	**0.6 ± 0.04** *	-	**0.7 ± 0.10** *	-
Paclobutrazol	0.016	0.8 ± 0.2	-	1.2 ± 0.15	0.8 ± 0.10	-	0.8 ± 0.10	-	1.0 ± 0.07	-
	0.08	0.9 ± 0.03	-	1.2 ± 0.15	0.8 ± 0.10	-	1.1 ± 0.20	-	1.0 ± 0.03	-
	0.4	0.9 ± 0.07	-	**1.6 ± 0.07** *	0.8 ± 0.20	-	0.8 ± 0.10	-	0.9 ± 0.08	-
Imazalil	0.04	0.8 ± 0.14	-	0.9 ± 0.08	**1.5 ± 0.10** *	0.9 ± 0.20	1.1 ± 0.13	-	0.9 ± 0.11	-
	0.2	0.8 ± 0.03	-	0.9 ± 0.08	**1.5 ± 0.07** *	**0.6 ± 0.03** *	1.1 ± 0.26	-	1.0 ± 0.08	-
	1	**0.7 ± 0.04** *	-	**2.5 ± 0.25** *	1.4 ± 0.23	0.9 ± 0.05	**2.0 ± 0.39** *	-	1.0 ± 0.18	-
Propiconazole	0.04	0.8 ± 0.03	-	**1.6 ± 0.33** *	0.8 ± 0.17	**0.5 ± 0.12** *	1.0 ± 0.06	-	0.9 ± 0.25	-
	0.2	0.8 ± 0.09	-	1.2 ± 0.26	0.9 ± 0.08	1.1 ± 0.20	1.0 ± 0.08	-	0.9 ± 0.06	-
	1	**0.7 ± 0.17** *	-	**1.8 ± 0.20** *	1.0 ± 0.08	**0.6 ± 0.11** *	1.2 ± 0.11	-	1.0 ± 0.11	-
L-AOPP	0.04	**0.5 ± 0.03** *	-	1.0 ± 0.08	**0.7 ± 0.07** *	-	**0.7 ± 0.09** *	**0.7 ± 0.11** *	1.1 ± 0.13	-
	0.2	**0.6 ± 0.15** *	-	1.2 ± 0.12	**0.7 ± 0.12** *	-	**0.7 ± 0.03** *	**0.5 ± 0.08** *	1.2 ± 0.12	-
	1	**0.6 ± 0.10** *	-	**1.8 ± 0.19** *	**0.7 ± 0.13** *	-	0.9 ± 0.14	**0.5 ± 0.06** *	1.3 ± 0.28	-
PEO-IAA	0.04	**0.7 ± 0.05** *	-	1.1 ± 0.13	0.8 ± 0.11	-	0.8 ± 0.08	**0.7 ± 0.05** *	1.1 ± 0.19	-
	0.2	**0.6 ± 0.08** *	-	1.3 ± 0.33	0.8 ± 0.14	-	**0.7 ± 0.06** *	**0.6 ± 0.07** *	1.3 ± 0.16	-
	1	**0.6 ± 0.04** *	-	1.4 ± 0.27	**0.7 ± 0.11** *	-	0.9 ± 0.09	**0.6 ± 0.10** *	0.9 ± 0.15	-
AIB	0.2	0.9 ± 0.13	-	**1.8 ± 0.27** *	1.1 ± 0.13	-	1.1 ± 0.27	-	**0.8 ± 0.04** *	**0.7 ± 0.04** *
	1	1.0 ± 0.13	-	**1.5 ± 0.07** *	**1.6 ± 0.16** *	-	1.1 ± 0.27	-	**0.8 ± 0.09** *	0.9 ± 0.10
	5	1.0 ± 0.18	-	**1.6 ± 0.16** *	**1.6 ± 0.31** *	-	**1.6 ± 0.25** *	-	1.1 ± 0.19	0.9 ± 0.06
AgNO_3_	0.12	**0.7 ± 0.07** *	-	**1.6 ± 0.21** *	1.0 ± 0.06	-	1.3 ± 0.17	-	**0.8 ± 0.08** *	0.9 ± 0.05
	0.6	1.0 ± 0.13	-	**2.1 ± 0.16** *	**2.5 ± 0.49** *	-	1.3 ± 0.05	-	0.9 ± 0.10	1.0 ± 0.04
	3	1.3 ± 0.19	-	**4.6 ± 0.48** *	**2.8 ± 0.35** *	-	1.4 ± 0.22	-	**1.7 ± 0.08** *	1.0 ± 0.17

Values are Mean ± SE (n = 6). Relative gene expression values setting UTC as 1.

* indicates significance at *P ≤ 0*.*05*.

In the case of Paclobutrazol, a GA synthesis inhibitor, the expression levels of *GA20ox2* [2], and other plant hormone responsive genes, as *OsbZIP23* (ABA), *DWF4* (BR), *OsIAA4* (Auxin), and *OsETR2* (Ethylene) were evaluated (Table 3). *GA20ox2* expression was up-regulated by increasing Paclobutrazol concentrations, while the expression of *OsbZIP23*, *DWF4*, *OsIAA4*, and *OsETR2* did not change significantly (Table 3).

The expression levels of the BR-responsive genes *DWF4* and *BU1* were evaluated following the application of BR inhibitors. In aerial parts, the up-regulation of *BU1* [43] and the down-regulation of *DWF4* [34, 44], following the application of BR, has been reported. *DWF4* expression level was up-regulated when the lowest and intermediate concentrations of Imazalil were applied (Table 3), and the expression of *BU1*, which increased with BR, was down-regulated by intermediate Imazalil concentration and the lowest Propiconazole concentration (Table 3). The expression of other plant hormone responsive genes, including *OsbZIP23*, *GA20ox2*, *OsIAA4*, and *OsETR2*, were analyzed. Only when the highest concentration of Imazalil and Propiconazole, and the lowest concentration of Propiconazole were applied, decreased expression of *OsbZIP23* and increased expression of *GA20ox2* were observed (Table 3). Our results would suggest that a specific inhibition of BR synthesis-related transcripts is elicited with either low or intermediate concentrations of Imazalil.

The expression levels of *OsIAA4* and *LOC_Os11g32110*, which were specifically increased by auxin [18, 45], and other plant hormone responsive genes were evaluated when the plants were treated with auxin inhibitors. PEO-IAA and L-AOPP reduced the expression of *LOC_Os11g32110*, while the expression of *OsIAA4* decreased with intermediate concentrations of PEO-IAA and L-AOPP (Table 3). PEO-IAA and L-AOPP also induced the down-regulation of *OsbZIP23* expression.

The effects of the ethylene inhibitors AIB and AgNO3 on the expression levels of *OsETR2* and *OsERS1*, which were specifically induced by ethylene [18, 46], and other plant hormone responsive genes (*OsbZIP23*, *GA20ox2*, *DWF4*, and *OsIAA4)* were evaluated (Table 3). Low AIB concentrations induced a decrease in *OsETR2* and *OsERS1* expression, while the expression of *GA20ox2* increased under both AIB and AgNO3 applications. The expression of *DWF4* was up-regulated at intermediate and high AIB and AgNO3 (Table 3). Thus, low concentrations of AIB inhibited the expression of genes associated with ethylene biosynthesis, and also affected GA-associated genes.

### NDGA and AIB improved photosynthesis and C assimilation

**Fig 1:**
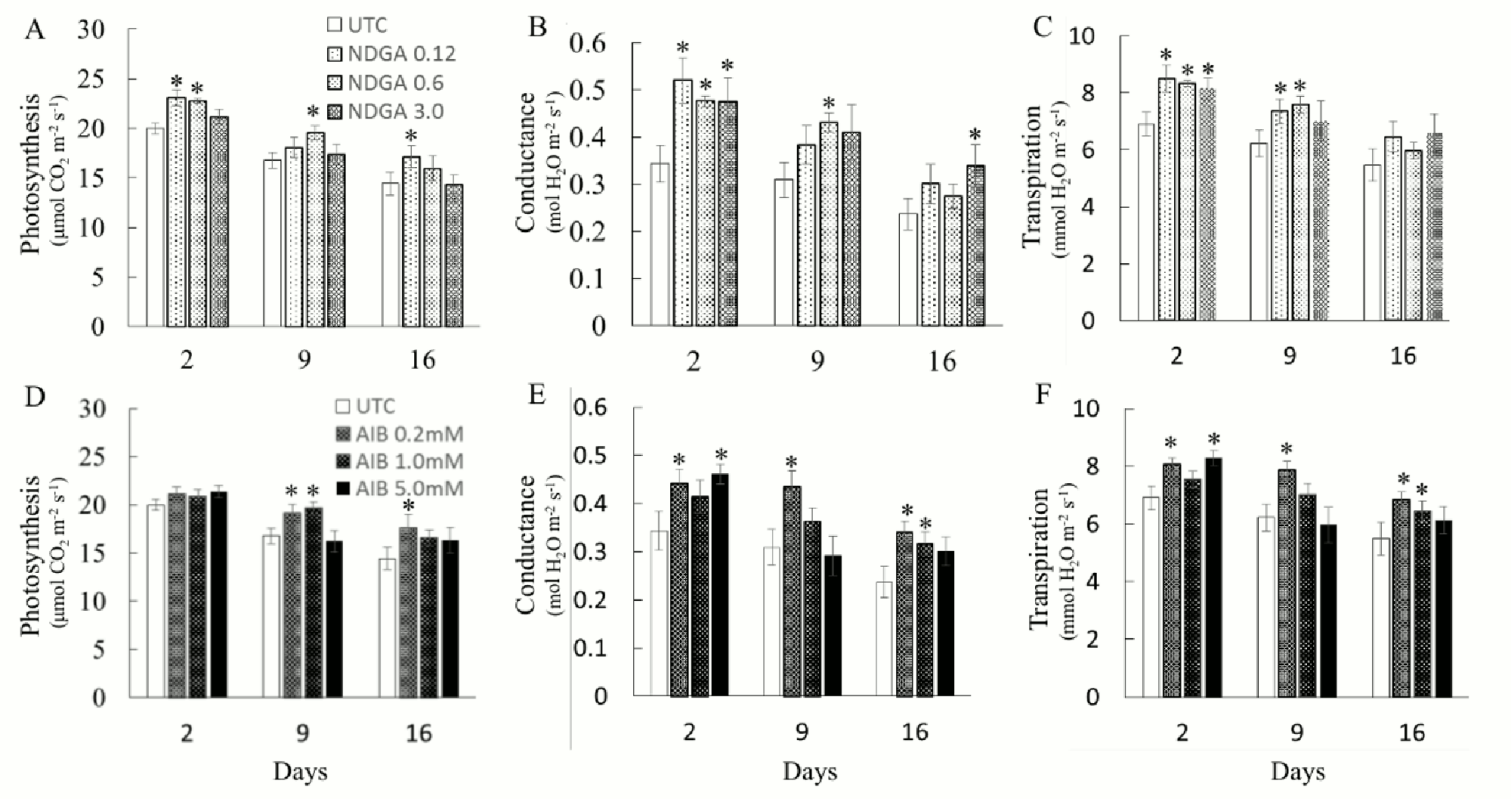
Effects of ABA and Ethylene biosynthesis inhibitors on photosynthesis, conductance, and transpiration rates at pre-anthesis. Photosynthesis rate (A, D), Conductance (B, E), and Transpiration rate (C, F) of UTC and NDGA treated plants (A, B, C) or UTC and AIB treated plants (D, E, F) 2, 9, and 16 d after spray at pre-anthesis stage. Values are Mean ± SE (n = 6). * indicating significance at *P ≤ 0*.*05*.

Photosynthesis and transpiration rates of rice flag leaves improved significantly 2 d after application of low NDGA concentration (Fig 1A and 1C). Higher transpiration and photosynthetic rates were maintained 9 and 16 d after treatment, respectively (Fig 1A and 1C). When NDGA was applied at the intermediate and higher concentrations, transpiration and conductance were significantly improved 2 d after application (Fig 1B and 1C). The low and intermediate AIB concentrations caused improvements on the photosynthetic and transpiration rates (Fig 1D and 1F). AIB treatments also improved photosynthesis and transpiration rate when applied at post-anthesis (S6 Fig). These results suggested that treatments with NDGA or AIB can increase the photosynthetic activity and leaf transpiration rates leading to increases of GY and SDW.

Highest paclobutrazol concentrations, decreased photosynthesis, stomatal conductance, and transpiration rates in flag leaves (S4 Fig), while Imazaril and PEO-IAA had no effect on these parameters (S5 Fig).

**Fig 2:**
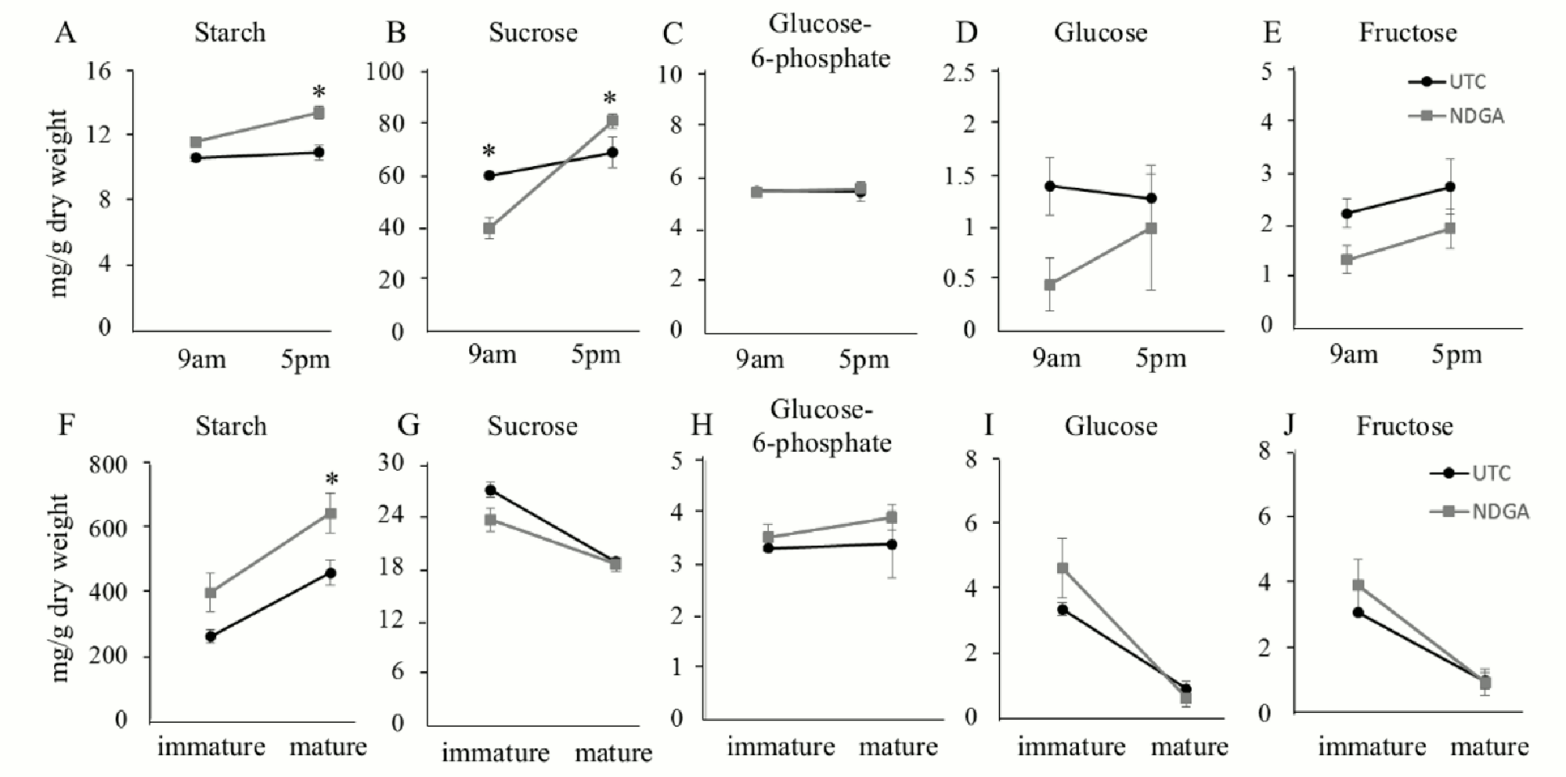
Starch and sugars contents in source and sink tissues treated with ABA biosynthesis inhibitor. Starch (A,F), Sucrose (B,G), Glucose-6-phosphate (C,H), Glucose (D,I), Fructose (E,J) contents in flag leaves (A-E) and immature/mature grains (F-J) treated with UTC and NDGA at pre-anthesis stage. Values are Mean ± SE (n = 4). * indicates significance at *P ≤ 0*.*05*.

Photosynthetic activity was shown to be positively correlated with the carbohydrate contents of flag leaves and grains [28, 47, 48]. In order to assess the effects of hormone inhibition on photosynthesis and the implications in carbon metabolism, we analyzed carbohydrate contents in flag leaves and grains of rice after NDGA or AIB application. Flag leaf samples treated with 0.12 mM NDGA at pre-anthesis displayed significantly higher starch and sucrose contents at the end of the day, as compared to the untreated controls (UTC). (Fig 2A and 2B) On the other hand, when the leaf samples were collected in the early morning (before the transitory starch starts to accumulate in leaves [49, 50]), no differences in starch content were found between UTC and 0.12 mM NDGA-treated samples (Fig 2A). These results were well correlated with the higher photosynthetic rates shown by NDGA-treated plants at pre-anthesis (Fig 1A). Starch and Sucrose contents of flag leaves were higher at the end of the day compared to early morning contents, but the increase was markedly larger in NDGA-treated plants (Fig 2A and 2B). No differences in glucose-6-phosphate (Fig 2C) and fructose contents (Fig 2E) were seen between NDGA-treated and UTC plants during the day. While the glucose contents of UTC plants at the early morning were similar to that at the end of the day (Fig 2D), the glucose content of flag leaves from NDGA-treated plants was lower than that of UTC plants at the early morning and increased at the end of the day to UTC levels. Starch content in mature grains was higher than immature grains in both NDGA-treated and UTC plants (Fig 2F). Sucrose, glucose and fructose contents in immature grains of UTC and NDGA-treated plants were higher compared to those of mature seeds (Fig 2G, 2I and 2J), while the glucose-6-phosphate contents of immature and mature grains from both NDGA and UTC plants were similar (Fig 2H).

**Fig 3:**
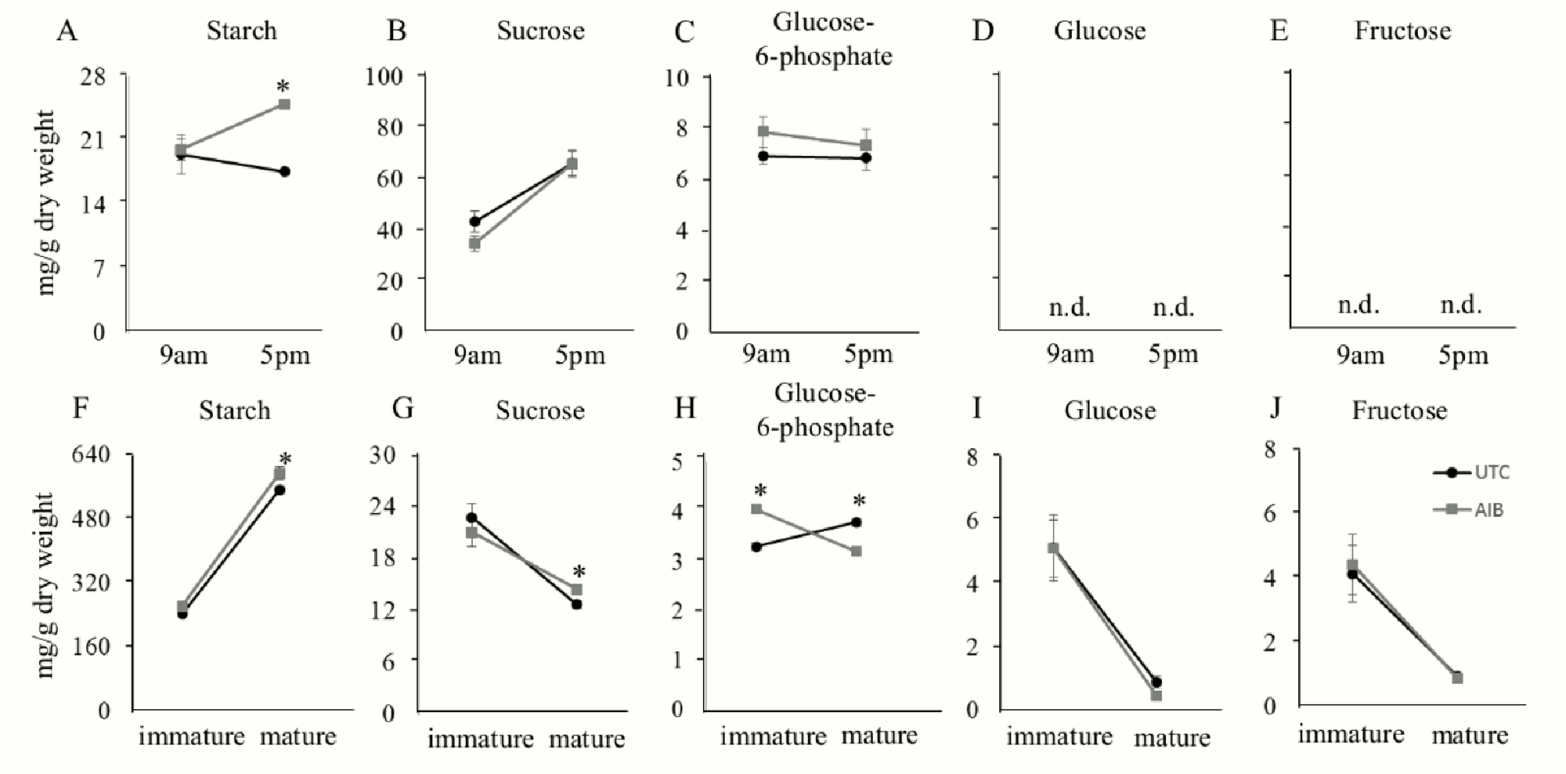
Starch and soluble sugars contents in source and sink tissues treated with Ethylene biosynthesis inhibitors. Starch (A,F), Sucrose (B,G), Glucose-6-phosphate (C,H), Glucose (D,I), Fructose (E,J) contents in flag leaves (A-E) and immature/mature grains (F-J) treated with UTC and AIB at post-anthesis stage. Values are Mean ± SE (n = 4). * indicates significance at *P ≤ 0*.*05*; n.d. indicates not detected.

Flag leaves from plants treated with 0.2 mM AIB at post-anthesis displayed higher starch content than the UTC plants at the end of the day, while starch content in flag leaves were similar to those from UTC plants at the early morning (Fig 3A). Flag leaves sucrose contents increased during the day, but there were no differences between AIB-treated and UTC plants (Fig 3B). In flag leaves of both AIB-treated and UTC plants, glucose-6-phosphate contents remained unchanged during the day (Fig 3C). Glucose and fructose contents could not be detected in plants treated with the inhibitor or in UTC plants (Fig 3D and 3E), a results that is consistent with sugar remobilization processes to sinks (grains) occurring at the end of plant life cycle [51]. Starch contents in mature seeds from AIB-treated and UTC plants were higher than those found in immature seeds (Fig 3F), while the sucrose content from both AIB-treated and UTC plants were higher in immature seeds than in mature seeds. No large differences were seen between AIB-treated and UTC plants in starch or sucrose contents (Fig 3G). Glucose-6-phosphate contents in AIB treated rice was higher or lower compared to UTC rice in immature or mature grains, respectively (Fig 3H). Fructose and glucose contents were higher in immature grains when compared to the contents found in mature grains from both AIB-treated and UTC plants (Fig 3I and 3J). These results suggested that the increase in photosynthetic activity by the NDGA and AIB applications can lead to increases of starch and/or sucrose in flag leaves, resulting in the increment of the starch content in mature grains of the NDGA and AIB treated plants.

In order to confirm whether the lowest NDGA concentration (0.12mM) inhibited ABA synthesis specifically, we quantified the contents of ABA, indole-3-acetic acid (IAA), GA1, GA4, trans-Zeatin (tZ), 2-isopentenyladenosine (iP), jasmonic acid (JA), jasmonic acid-Isoleucine (JA-Ile), and salicylic acid (SA). JA and JA-Ile contents decreased significantly after the application of 0.12 mM NDGA (Table 4). Although the down-regulation of ABA-related transcripts was observed after the applicaton of 0.12 mM NDGA, ABA contents were not significantly reduced in the 0.12 mM NDGA-treated plants. Also, IAA, GAs, Cytokinins, and SA did not change significantly with 0.12mM NDGA treatment (Table 4). In the AIB-treated rice plants, no significant differences in plant hormone contents were found when compared to UTC rice (Table 4).

**Table 4:** Plant hormone contents in NDGA and AIB treated rice.

Hormone content (ng/gDW)	UTC	NDGA	UTC	AIB
ABA	117.5 ± 11.0	107.8 ± 11.6	90.0 ± 20	76.2 ± 12
IAA	21.3 ± 2.1	18.3 ± 1.6	36.2 ± 8.5	32.1 ± 6.5
GA_1_	0.56 ± 0.34	0.9 ± 0.03	n.d.	n.d.
GA_4_	0.4 ± 0.2	0.5 ± 0.06	0.8 ± 0.5	2.6 ± 1.1
tZ	1.4 ± 0.2	1.5 ± 0.1	0.5 ± 0.05	0.32 ± 0.06
iP	0.2 ± 0.02	0.18 ± 0.01	0.33 ± 0.02	0.42 ± 0.08
JA	74.6 ± 14.8	**42.8 ± 6.2** *	5.2 ± 0.5	5.3 ± 0.9
JA-Ile	30.2 ± 5.9	**13.5 ± 2.4** *	2.6 ± 0.5	1.7 ± 0.7
SA (μg/gDW)	34.7 ± 3.3	28.7 ± 1.1	20.2 ± 1.2	17.8 ± 1.2

0.12mM NDGA was applied at pre-anthesis and 0.2mM AIB was applied at post-anthesis. Values are Mean ± SE (n = 5 or 4). DW, dry weight.

* indicates significance at *P ≤ 0*.*05*. n.d. indicates not detected.

**Fig 4:**
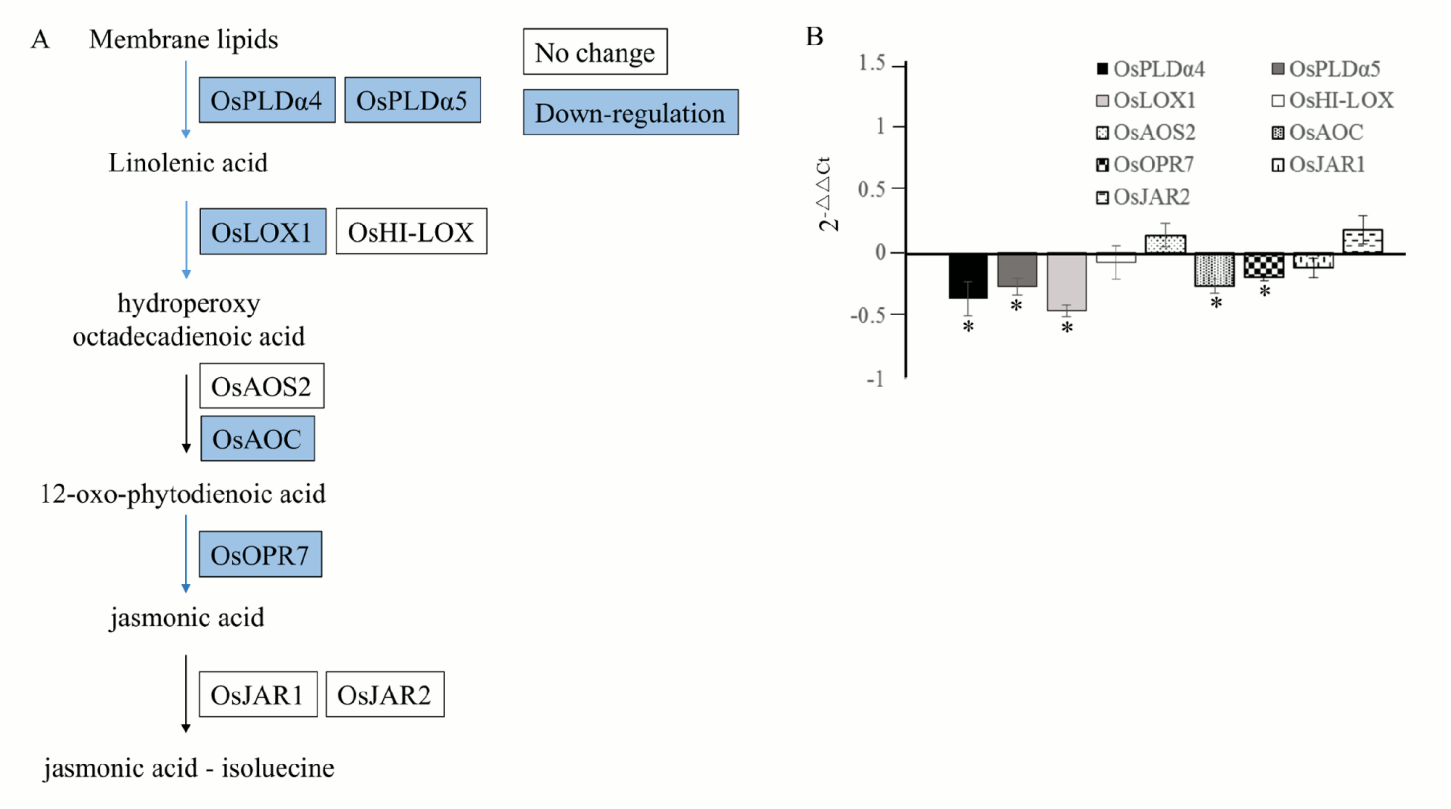
Effects of NDGA on the expression of JA synthesis-related genes. Effects of NDGA on the expression of JA synthesis-related genes (*OsPLDα4* [LOC_Os06g40170], *OsPLDα5* [LOC_Os06g40180], *OsLOX1* [LOC_Os03g49380], *OsHI-LOX* [LOC_Os08g39840], *OsAOS2* [LOC_Os03g12500], *OsAOC* [LOC_Os03g32314], *OsOPR7* [LOC_Os08g35740], *OsJAR1* [LOC_Os05g50890] and *OsJAR2* [LOC_Os01g12160]) 2 days after treatment at pre-anthesis stage. (A) Scheme of the main steps in the JA synthesis pathway indicating (in colored boxes) the genes that were differently expressed in NDGA treated plants with respect to UTC plants. *White* indicates genes not significantly different and *Blue* indicates genes with a significantly lower gene expression. (B) Relative gene expression values setting UTC as 0. Values are Mean ± SE (n = 6). * indicating significance at *P ≤ 0*.*05*.

We also evaluated the effects of 0.12 mM NDGA on the expression of JA synthesis-related genes including, rice *Phospholipase D alpha 4* (*OsPLDα4)* [LOC_Os06g40170], rice *Phospholipase D alpha 5 (OsPLDα5)* [LOC_Os06g40180], rice *lipoxygenases* (*OsLOX1* [LOC_Os03g49380] and *OsHI-LOX* [LOC_Os08g39840]), rice *allen oxide synthase* (*OsAOS2* [LOC_Os03g12500]), rice *allen oxide cyclase* (*OsAOC* [LOC_Os03g32314]), *rice 12-oxophytodienoate reductase 7* (*OsOPR7* [LOC_Os08g35740]), rice *jasmonic acid resistance 1 and 2* (*OsJAR1* [LOC_Os05g50890] and *OsJAR2* [LOC_Os01g12160]) 2 d after treatment at pre-anthesis ([52] and references therein). The decreased expression of *OsPLDα4*, *OsPLDα5*, *OsLOX1*, *OsAOC*, and *OsOPR7*, following the NDGA treatment, suggested the NDGA-induced inhibition of the JA synthesis pathway (Fig 4). These results indicated that the repression of JA synthesis-related genes expressions might contribute to the reduction of JA and JA-Ile contents.

## Discussion

In this study we analyzed the effects of hormone biosynthesis/signaling inhibition at reproductive stage on grain yield and plant biomass in rice. The expression analysis of different hormone responsive genes allowed determining the inhibitor concentrations that down-regulate specific plant hormone pathways. Also, the correlation between the hormone inhibition and alterations in C assimilation, GY and plant biomass, could contribute to the targeting of pathways to improve cereal crops productivity.

### Effects of hormone inhibition on plant biomass

Plant hormones play important roles in the regulation of plant growth and development and in the plant response(s) to changes in the environment [6, 7, 53].

GA is known to regulate plant growth and grain yield [54, 55]. Paclobutrazole and uniconazole were reported to inhibit GA biosynthesis based on the analysis of GA-associated phenotypes, GA content measurements (before and after treatments) and by the expression analysis of *GA20ox2* [37, 56]. When high Paclobutrazol concentrations (0.28kg/ha = 28mg/m2) were applied at pre-anthesis in rice grown under field conditions, GY was decreased [21]. In the present study, Paclobutrazol treatments of 18.3mg/ m2 or 91.4mg/ m2 (S1 Table), caused a GY decrease of 20% and 24%, respectively (Table 1). Since, the expression of genes associated with other hormones remained unchanged, the Paclobutrazol-induced decrease in GY could be considered to be exclusively associated with its inhibitor effect on GA biosynthesis.

The overexpression of *DWF4*, a gene encoding a cytochrome P450 that mediates BR biosynthesis in plants, was shown to improve GY in rice transgenic plants grown in the field [57]. In *Arabidopsis*, Imazalil was reported to inhibit the growth of hypocotyls and roots in a concentration dependent manner through the regulation of *DWF4* [33]. Our results showed that intermediate concentrations of Imazalil reduced *BU1* expression [43] and up-regulated *DWF4* expression, which has been shown to be down-regulated by increased BR levels [34, 42]. The effects of intermediate concentrations of Imazalil appeared to specifically affect BR biosynthesis, since the expression of other hormone-responsive genes remained unchanged (Table 3). BRs have been associated with important roles controlling plant biomass and GY [58–61]. Although it has been reported that mutations in *DWF4* improved GY in rice [61], the ectopic overexpression of *DWF4* resulted in the improvement of GY in *Arabidopsis*, rice and cotton [62]. Our results (Tables 1 and 3) indicated that the increase in *DWF4* expression did not result in increased plant biomass or GY, thus highlighting the importance of developmental stage and tissue-specificity in BR regulation. Propiconazole has been shown to inhibit specifically BR synthesis, limiting the growth of *Arabidopsis* and Maize in a concentration dependent manner [34]. A significant decrease in shoot biomass occurred when Propiconazole was applied (Tables 1 and 2). Contrary to the results previously shown [34], *GA20ox-2* expression was affected by propiconazole application and by high concentrations of imazalil (Table 3) suggesting a possible crosstalk between BR and GA biosynthesis in rice. Interestingly, a mechanism of crosstalk between BR and GA pathways has been identified ([63] and references therein).

In barley and *Arabidopsis*, a steeped auxin reduction by high temperature caused the abortion of pollen development and a decreased GY [64]. In rice, auxin deficiency resulted in severe growth retardation at the early vegetative stage [65]. To test the effects of auxin deficiency at the reproductive stage, we used L-AOPP (an inhibitor of Auxin biosynthesis [31]), and PEO-IAA (a TIR1 (Transport Inhibitor Response 1)-dependent auxin receptor inhibitor [66]). The low and intermediate concentration of L-AOPP inhibited auxin responses (Table 3), and decreased GY by 15% and 20%, respectively (Tables 1 and 2), suggesting that auxin deficiency at the reproductive stage has also a negative impact on growth. In rice, the over-expression of *OsGH3-2*, a gene encoding an auxin-conjugating enzyme, resulted in auxin deficiency leading to a significant down-regulation of ABA-related genes such as *OsbZIP23* [67]. We observed that all L-AOPP and PEO-IAA concentrations reduced the expression levels of *OsbZIP23* (Table 3), indicating a L-AOPP inhibition of auxin biosynthesis similar to that reported in *OsGH3-2* overexpression lines [67]. Auxin binds to two types of receptors designated as *AUXIN BINDING-PROTEIN 1* (ABP1), which regulates clathrin-dependent vesicle transport, and *TRANSPORT INHIBITOR RESPONSE 1* (TIR1), that regulates auxin-dependent transcription [68–71]. PEO-IAA is an antagonist on TIR1 Auxin receptor [66]. Our results suggested that auxin-dependent transcription through TIR1 regulation, modified OsbZIP23 expression in rice.

Our results show that ethylene biosynthesis inhibitor increased GY and SDW in rice as is the case with ethylene perception inhibitors [22, 23]. The lowest concentration of AIB reduced the expression levels of *OsETR2* and *OsERS1*, and induced *GA20ox2* expression (Table 3). *GA20ox2* is involved in GA biosynthesis and the loss of GA20ox2 function was associated with dwarf rice phenotypes and increased GY [72, 73]. On the other hand, ethylene regulates GA content and signaling, but this regulation is dependent on ethylene concentrations, the plant developmental stage and the environmental conditions [74, 75]. This complex regulation may explain the variation observed in the *GA20ox2* expression levels among the different AIB concentrations used in this study.

NDGA inhibits the function of a lipoxygenase required for ABA biosynthesis [76, 77]. It was also shown that NDGA inhibited ABA biosynthesis in some fruits during fruit maturation period [24, 78], and in maize, soybean, and rice under drought stress conditions [30, 77, 79]. Nevertheless, effects of NDGA on GY have not yet been reported. Our results indicated that that lowest NDGA concentration did not affect the expression levels of any plant hormone-responsive genes other than *LEA3*, but resulted in a significant increase of rice GY (21%) (Tables 1 and 3). In contrast, the intermediate NDGA concentration decreased GY by 16%, reduced the expression levels of *OsbZIP23* and *LEA3*, and promoted the expression level of *GA20ox2* (Tables 1–3). It has been shown that ABA inhibits GA biosynthesis through the transcriptional regulation of GA biosynthesis-related genes including GA20ox during germination [38]. Based on this evidence, we could assume that the intermediate concentration of NDGA might reduce ABA content, causing the up-regulation of *GA20ox2* at the reproductive stage in rice. The *GA20ox2* up-regulation was comparable to the gene induction observed when the high concentration of Paclobutrazol treatment was used (Table 3).

### Inhibition of jasmonic acid and ethylene biosynthesis improved photosynthesis promoting changes in C metabolism

In rice, the flag leaves are the main source of photoassimilates that will contribute rice grain filling [80, 81]. Thus, flag leaf photosynthesis can be considered one of the main metabolic pathways determining grain filling in grains and flag leaf photosynthetic activity is directly correlated with grain yield [82–84].

When applied at pre-anthesis stage, NDGA improved photosynthesis and transpiration rates (Fig 1A and 1C), and promoted transitory starch accumulation in flag leaves (Fig 2A). Transitory starch will be converted to sucrose in the leaves and subsequently translocated into the grains during grain filling, thus transitory starch content in leaves at pre-anthesis stage is critical to determine grain carbohydrate content [80, 85]. The increase in grain starch content observed after NDGA treatment was correlated with starch accumulation in flag leaves (Fig 2A and 2F). Gene expression analysis also revealed that ABA responsive genes were down-regulated (Table 3) but this reduction was not correlated with a significant decrease in ABA content (Table 4). Interestingly, 0.12mM NDGA significantly decreased JA and JA-Ile contents (Table 4), and the expression of some JA synthesis-related genes (Fig 4). OsPLDα4 and OsPLDα5 mediate the release of linolenic acid from membrane phospholipids in the chloroplast [86] and LOX oxidize linolenic acid to generate hydroperoxy octadecadienoic acid (HPODE) [87]. HPODE is then converted to 12-oxo-phytodienoic acid (OPDA) by OsAOS and OsAOC. OsOPR7 generates jasmonic acid from OPDA and OsJAR1 / OsJAR2 catalyze the final step to form bioactive JA and, JA-Ile [88]. Among of these genes, the expressions of *OsPLDα4*, *OsPLDα5*, *OsLOX1*, *OsAOC*, and *OsOPR7* were significantly decreased by the 0.12mM NDGA application (Fig 4). The reduced expression of *OsPLDα4* / *OsPLDα5*, and a mutation in *OsAOC* were correlated with the decrease of JA contents in rice, altering plant defense responses [86, 89]. Thus, it is possible that the decreased expression of *OsPLDα4*, *OsPLDα5*, and *OsAOC* results in JA and JA-Ile decreased content in rice leaves followed by NDGA application. Similar to ABA [90], the exogenous application of JA inhibited photosynthesis by promoting stomata closure [91, 92], resulting in grain yield penalties in rice and soybean [15, 92]. Thus, we can assume that the inhibition of JA synthesis might be responsible for the grain yield improvement observed after NDGA application.

Ethylene has been shown to inhibit photosynthesis in peanut, sunflower, and sweet potato by promoting stomata closure [93], and the inhibition of ethylene synthesis and/or perception improved GY under stressed conditions in rice [94]. Yet, the mechanism(s) by which ethylene inhibition acts in a time-dependent manner on photosynthesis regulation remains unclear. In order to assess the effect of ethylene regulation on grain yield and plant biomass, AIB was applied at two different time points during the reproductive stage (pre- and post-anthesis). When the lower AIB concentration was applied at pre-anthesis, no improvement of GY and a decreased SDW were observed (Table 1). However, when AIB was applied at post-anthesis, an improvement of GY (22%) and SDW (21%) was detected (S4 Table). Furthermore, AIB improved photosynthesis and transpiration (Fig 1D, 1F and S6 Fig), and these were well correlated with increased starch content in the flag leaves and mature grains in AIB-treated plants (Fig 3A and 3F). Ethylene inhibition seems to be beneficial for photosynthesis and C-metabolism in flag leaves. Nevertheless, ethylene play important roles during flower development, regulating the stimulation of gametophyte development and pollen tube growth [95, 96]. Ethylene inhibition at pre-anthesis also inhibits proper flower development in rice [97], thus the development-specific regulation of ethylene might be critical to determine GY during rice development.

Our data supports the notion that the inhibition of JA or ethylene at reproductive stage improved grain yield in rice. The specific inhibition of GA, BR or auxins, negatively affected GY and plant biomass while the inhibition of JA or ethylene resulted in higher GY and plant Biomass through the improvement of photosynthesis, C-assimilation and sugar mobilization to the sinks.

## Supporting Information

S1 Fig Phenotypes of plants treated with plant hormone inhibitors. Plant Phenotypes of UTC (A), NDGA (B), Paclobutrazol (C), Imazalil (D), Propiconazole (E), L-AOPP (F), PEO-IAA (G), AIB (H), and AgNO3 (I) treated rice 9 days after spray at pre-anthesis. Each figure shows the plants sprayed with either the lowest (L), intermediate (M), or the highest (H) concentration (mM) of each chemical treatment. Bar = 20 cm.
(TIF)

S2 Fig Rice grain phenotypes of grains of plants treated with plant hormone inhibitors at pre-anthesis. Grains of either UTC (A), NDGA (B), Paclobutrazol (C), Imazalil (D), Propiconazole (E), L-AOPP (F), PEO-IAA (G), AIB (H), or AgNO_3_ (I) treated rice sprayed at pre-anthesis. Each figure shows the grains harvested from the rice treated with either the lowest (L), intermediate (M), or the highest (H) concentration (mM) of each chemical treatment. Bar = 1.5 cm.
(TIF)

S3 Fig Rice grain phenotypes of grains of plants treated with plant hormone inhibitors at both pre- and post-anthesis. Grains of either UTC (A), NDGA (B), Paclobutrazol (C), Imazalil (D), Propiconazole (E), L-AOPP (F), PEO-IAA (G), AIB (H), or AgNO_3_ (I) treated rice sprayed at both pre-anthesis and post-anthesis. Each figure shows the grains harvested from the rice treated with either the lowest (L), intermediate (M), or the highest (H) concentration (mM) of each chemical treatment. Bar = 1.5 cm.
(TIF)

S4 Fig Effect of GA synthesis inhibitor on photosynthesis, conductance, and transpiration rate at pre-anthesis stage. Photosynthesis (A), Conductance (B), and Transpiration rates (C) of UTC and Paclobutrazol treated plants 2, 9, and 16 days after treatment at pre-anthesis stage. Values are Mean ± SE (n = 6). * indicates significance at *P ≤ 0*.*05*.
(TIF)

S5 Fig Effect of BR synthesis inhibitor and Auxin perception inhibitor on photosynthesis, conductance, and transpiration rates at pre-anthesis stage. Photosynthesis (A, D), Conductance (B, E), and Transpiration rates (C, F) of UTC and Imazalil treated plants (A, B, C) or UTC and PEO-IAA treated plants (D, E, F) 2, 9, and 16 days after treatment at pre-anthesis stage. Values are Mean ± SE (n = 6). No significant difference among treatments.
(TIF)

S6 Fig Effect of Ethylene synthesis inhibitors on photosynthesis, conductance, and transpiration rates at post-anthesis stage. Photosynthesis (A), Conductance (B), and Transpiration rates (C) of UTC and AIB treated plants 2 and 9 days after treatment at post-anthesis stage. Values are Mean ± SE (n = 6). * indicates significance at *P ≤ 0*.*05*.
(TIF)

S1 Table Plant hormone inhibitors used in this study. (XLSX)

S2 Table LC conditions. (DOCX)

S3 Table Parameters for LC-ESI-MS/MS analysis. (DOCX)

S4 Table Time-dependent effects of plant hormone inhibitors on plant biomass production and grain yield. Values are Mean ± SE (n = 6). * indicates significance at *P ≤ 0*.*05*.
(XLSX)

S5 Table List of primer sequences used in quantitative PCR (Real Time qPCR) assays in this work. (XLSX)
