# Supplementary material for: Targeting Hormone-Related Pathways to Improve Grain Yield in Rice: A Chemical Approach
Source: PLoS One. 2015 Jun 22;10(6):e0131213. doi: 10.1371/journal.pone.0131213 (PMC4476611; doi:10.1371/journal.pone.0131213)
Supplement: S2 Table — (DOCX) [file pone.0131213.s008.docx]

| **No** | **Solvent A** | **Solvent B** | **Gradient (composition of solvent B)** |
| --- | --- | --- | --- |
| **1** | **Water containing 0.01% acetic acid** | **MeCN, 0.05 % acetic acid** | **3 to 50 % over 15 min** |
| **2** | **Water containing 0.1% formic acid** | **MeCN, 0.1 % formic acid** | **3 to 69.5 % over 7 min** |
| **3** | **Water containing 0.01% acetic acid** | **MeCN, 0.05 % acetic acid** | **3 to 16.8 % over 20min** |

**S2 Table. LC conditions.**
