# Supplementary material for: Targeting Hormone-Related Pathways to Improve Grain Yield in Rice: A Chemical Approach
Source: PLoS One. 2015 Jun 22;10(6):e0131213. doi: 10.1371/journal.pone.0131213 (PMC4476611; doi:10.1371/journal.pone.0131213)
Supplement: S3 Table — (DOCX) [file pone.0131213.s009.docx]

| **LC conditions** | **ESI** | **MS/MS** | **transitions for quantifications (m/z)** | **Collision energy　(V)** | **Fragmentor　(V)** |
| --- | --- | --- | --- | --- | --- |
|  |  |  |  |  |  |
|  |  |  |  |  |  |
| **1** | **negative** | **GA_1_** | **347/273** | **23** | **150** |
|  |  | **D_2_-GA_1_** | **349/275** |  |  |
| **1** | **positive** | **IAA** | **176/130** | **13** | **90** |
|  |  | **D_2_-IAA** | **178/132** |  |  |
| **1** | **negative** | **ABA** | **263/153** | **3** | **105** |
|  |  | **D_6_-ABA** | **269/159** |  |  |
| **1** | **negative** | **JA** | **209/59** | **9** | **95** |
|  |  | **D_2_-JA** | **211/59** |  |  |
| **1** | **negative** | **GA_4_** | **331/257** | **21** | **140** |
|  |  | **D_2_-GA_4_** | **333/259** |  |  |
| **1** | **negative** | **JA-Ile** | **322/130** | **18** | **140** |
|  |  | **^13^C_6_-JAIle** | **328/136** |  |  |
| **2** | **negative** | **SA** | **137/93** | **13** | **85** |
|  |  | **D_6_-SA** | **141/97** |  |  |
| **3** | **positive** | **tZ** | **220/136** | **13** | **110** |
|  |  | **D_5_-tZ** | **225/136,137** |  |  |
| **3** | **positive** | **iP** | **204/136** | **10** | **100** |
|  |  | **D6-iP** | **210/137** |  |  |

**S3 Table. Parameters for LC-ESI-MS/MS analysis.**
